# Earlier hatching date rather than chick phenotypic quality enhances first-year survival in the black-headed gull

**DOI:** 10.1186/s12983-026-00605-6

**Published:** 2026-03-17

**Authors:** Piotr Indykiewicz, Julia Barczyk, Jarosław Kowalski, Piotr Minias

**Affiliations:** 1 Faculty of Animal Breeding and Biology, Department of Biology and Animal Environment, UTP University of Science and Technology, Mazowiecka 28, 85-084 Bydgoszcz, Poland; 2 Faculty of Biological Sciences, Department of Avian Ecology, University of Wrocław, Sienkiewicza 21, 50-335 Wrocław, Poland; 3 Gorzewo 7, 09-200 Sierpc, Poland; 4 Faculty of Biology and Environmental Protection, Department of Biodiversity Studies and Bioeducation, University of Lodz, Banacha 1/3, 90-237 Lódź, Poland

**Keywords:** Black-headed gull, Condition, Hatching date, Immune response, Post-fledging period, Survival rate

## Abstract

**Background:**

Favourable conditions experienced during early ontogeny are expected to positively influence individual performance at later stages of the life cycle and determine lifetime fitness. In this study, we investigated whether early-life (chick) phenotypic quality (body condition and immunocompetence) and hatching date are associated with post-fledging survival in the black-headed gull *Chroicocephalus ridibundus*. To this end, we quantified size-corrected body mass, total blood haemoglobin concentration, and PHA-induced immune response in more than 500 colour-marked gull chicks in Poland. We then analysed their first-year survival rates using capture-recapture models based on extensive resighting data collected across Europe.

**Results:**

Neither better body condition (body mass and haemoglobin concentration) nor higher immunocompetence during rearing significantly improved post-fledging (first year of life) survival rate. In contrast, post-fledging survival was primarily determined by hatching date, as early-hatched chicks showed enhanced survival rates than those hatched late in the season. The positive effect of early hatching on post-fledging survival was apparent despite lower phenotypic quality of those early-hatched chicks.

**Conclusions:**

Our study identifies hatching date as the key determinant of post-fledging survival in the black-headed gull. We suggest that the cumulative negative effects of delayed (suboptimal) hatching and fledging in black-headed gulls may outweigh any benefits of elevated condition and immunocompetence at the natal stage, ultimately compromising post-fledging survival. We propose that our findings may be explained by varying levels of temporal (mis)synchronization between peak food availability and key stages of the annual cycle, particularly the early post-fledging period. The study adds to the understanding of the carry-over effects between the successive stages of the life cycle in wild colonial birds.

**Supplementary Information:**

The online version contains supplementary material available at 10.1186/s12983-026-00605-6.

## Background

The early stages of life are widely recognized as crucial for lifetime fitness [1, 2]. Complex interactions between genetic makeup and the environment impose significant and long-lasting effects on phenotype (e.g. body size, physiological function, or behaviour), and consequently on survival and reproductive success [1, 3]. The environment influences young individuals both directly and indirectly. Indirect effects can occur through maternal effects [4, 5], including maternal depositions of egg components (e.g. nutrients, hormones, and antibodies) [6, 7], but also through environmental conditions during incubation [8–11]. In contrast, direct environmental influences during development include a wide array of factors, such as food availability [12], weather conditions [13], predation pressure [14, 15], parasitism [16] and exposure to environmental pollution [17]. These early-life conditions may have long-term consequences for individuals, particularly in long-lived species [3, 18].

According to the silver spoon hypothesis [19] individuals reared under favourable environmental conditions achieve greater success later in life than those reared in poorer conditions, regardless of the adult environment [1, 19]. For instance, individuals that experience advantageous early-life conditions often acquire better body condition, which can positively influence expression of condition-dependent traits later in life [3, 20, 21]. Early-life advantages can also modulate age of first recruitment to the population, fertility, and reproductive success, having overall positive effects on fitness [3, 18, 22–24]. Carry-over effects of early-life conditions are, though, expected to be complex and context-dependent [25]. For example, long-term effects of early-life environment can be sex-specific [26], change between life stages [27] or show local variation among populations [28]. Rearing conditions may also be strongly modulated by phenology, as the timing of development determines synchronization of key life stages with food availability [29]. Early breeding by birds in seasonal environments is traditionally thought to be promoted by natural selection, as it better matches the local peak in food abundance, thus enhancing reproductive success [30]. This temporal synchronization may also have long-term consequences for offspring. So far, early avian hatching dates were linked, among the others, to the occupancy of better-quality winter habitats [31], higher pre-migratory fat stores [32], higher reproductive investment during the first breeding [33], longer lifespan and higher lifetime reproductive success [34]. On the other hand, in some avian species early life phenotypic quality (e.g. immunocompetence) was identified as a better predictor of fitness than hatching date [e.g. 35].

Evidence for the effects of early life on survival in natural bird populations is accumulating. For example, the length of the rearing period was positively associated with local survival before recruitment in the kittiwake *Rissa tridactyla* [36]. Juvenile survival was also highly sensitive to early-life conditions, such as food availability per capita during rearing (Audouin’s gull *Larus audouinii* [2]) or natal habitat quality (oystercatcher *Haematopus ostralegus* [37]). In contrast, aggressive subordination, food deprivation and elevated stress hormones imposed by sibling conflict had no detectable effects on survival or recruitment rate in the blue-footed booby *Sula nebouxii* [38]. Post-fledging survival was also linked to hatching or fledgling date, although the directions of these associations varied between species and years [e.g. 39–42]. These contrasting patterns suggest that carry-over effects may be taxa-specific or context-dependent [43].

The primary aim of this study was to investigate the association of early-life phenotypic quality and hatching date with post-fledging survival in the black-headed gull *Chroicocephalus ridibundus* – a migratory, colonial species with high philopatry [44, 45]. Similarly to most larid species, black-headed gulls are relatively long-lived, with the maximum lifespan reaching 33 years in the wild [46]. However, from a life history perspective, the first year of life is expected to be particularly critical for black-headed gulls, as it overlaps with several demanding stages: becoming independent from parents, the first two moulting events (July–September and February–April), and the first migration/wintering periods. At each of these stages, young gulls are exposed to various environmental and social stressors (e.g. predation selectively targeted at more naïve young birds or competition with older more dominant conspecifics) and constraints (e.g. resource allocation trade-offs). We therefore hypothesized that phenotypic quality during the natal period may have a strong impact on future fitness, particularly post-fledging survival. To test this hypothesis, we measured body condition (size-corrected body mass and total blood haemoglobin concentration) and immunocompetence (induced immune response) in more than 500 black-headed gull chicks from Poland. We also collected extensive resighting data from our colour-marked individuals and analysed their post-fledging survival rates using capture-recapture models. Finally, because rearing conditions may vary considerably throughout the breeding season, we examined temporal (intra-seasonal) variation in chick condition and immunocompetence and tested for the effects of this variation on post-fledging survival.

## Methods

### Study sites and general field methods

The study was conducted from 2019 to 2022 in four breeding colonies of the black-headed gull in north-central and central Poland. The colonies were located on islands at: i) Koronowskie reservoir near the village of Pieczyska (53.3344 N, 17.9650 E), ii) Pakoskie reservoir near the village of Jankowo (52.7830 N, 18.0842 E), iii) artificial ponds in an urban zone of Bydgoszcz (53.1645 N, 18.0367 E), and *iv*) active gravel pit in Dąb Polski (52.6067 N, 19.3838 E). The size of the colonies ranged from 250 pairs (Bydgoszcz) to ca. 12 thousand pairs (Dąb Polski). In each colony, we carried out regular (2–3 times a week) nest surveys during the egg laying and incubation periods. After peak egg laying but before hatching of the first chicks, we constructed 3–5 nest enclosures (20–25 m^2^ each) in each colony to prevent chicks from leaving the monitored area during the repeated visits by researchers. Enclosures were constructed from 1.2 m high nets with 2 cm mesh and were randomly distributed throughout each colony, i.e. across the centre-periphery gradient and the range of microhabitats. The density of nests within the enclosures ranged from 0.6 to 1.4 nests/m^2^. Each chick hatched within the enclosures was marked with a metal alphanumeric ring (left tarsus) within two days of hatching. The peak hatching period was between late April and late May. We aimed to quantify phenotypic quality (body condition and immunocompetence, see below for details) in all the chicks within the enclosures, focusing mostly on individuals older than two weeks (median age = 18 days). All chicks subjected to measurements were additionally marked with a colour plastic alphanumeric ring on the right tarsus. Chicks that did not survive to fledging were excluded from further analyses. In total, we obtained phenotypic quality measurements from 530 colour-marked chicks that successfully fledged (79–195 chicks per colony).

### Chick body condition

Chick condition was measured using two complementary approaches. First, we quantified size-corrected body mass, which reflects nutritional status and absolute energy reserves, i.e. fat and protein [47]. For this purpose, we measured chick body mass (± 1 g) with an electronic balance and wing length (± 1 mm) with a stopped ruler. Because log–log regression of body mass against wing length was non-linear (91.9% vs. 79.2% of variance explained by quadratic and linear regression, respectively), we did not apply the scaled mass index recommended by Peig and Green [48]. Instead, we extracted residuals using the log-quadratic regression of body mass against wing length (henceforth referred to as size-corrected body mass). Extracted residuals were independent of chick age (Table S1).

Second, we assessed chick physiological condition using total blood haemoglobin concentration—a reliable proxy for blood oxygen-carrying capacity in birds (reviewed in Minias [49]). In general, blood haemoglobin concentration has been shown to correlate with nutritional condition, diet quality, and developmental stability in both adults and chicks [49]. Here, we determined chick blood haemoglobin concentration with the azide-methemoglobin method using a portable HemoCue Hb 201 + photometer (HemoCue, Ängeholm, Sweden), which provides accurate and repeatable measurements in avian blood [50, 51]. For the purpose of measurements, approximately 5 μl of blood was collected from the ulnar vein of each chick with a disposable needle and immediately transferred into a disposable HemoCue microcuvette. Absorbance, directly proportional to haemoglobin concentration, was measured within 5 min of sampling. Due to technical constraints (photometer malfunction), it was not feasible to measure haemoglobin concentration in 93 chicks, resulting in the final sample size of 437 haemoglobin measurements. The development of blood oxygen-carrying capacity in chicks typically follows a quadratic curve, rapidly increasing early during ontogeny and then quickly stabilizing [52]. In black-headed gull chicks, a quadratic curve relatively well described age-related variation in blood haemoglobin concentration measurements (28.0% of variance explained). Consequently, we extracted age-corrected (residual) blood haemoglobin concentration values from a quadratic regression and used them in the analysis.

### Induced immune response

To assess induced immune response in chicks we used the phytohaemagglutinin (PHA) skin test. The PHA has mitogenic and inflammatory properties, stimulating the aggregation of immune cells at the injection site and causing local swelling [53]. Although the immunological mechanisms underlying the PHA response are complex and involve a combination of innate and acquired immune pathways [54, 55], the PHA skin test has been widely used in ecoimmunological research on wild birds, primarily due to its technical simplicity [56–58]. Here, we quantified the PHA-induced immune response (henceforth PHA response) in black-headed gull chicks using disposable syringes with 0.33 mm gauge needles to inject 0.2 mg PHA (Phytohaemagglutinin PHA-P L8754, Sigma-Aldrich, St. Louis, MO, USA) dissolved in 0.05 ml phosphate-buffered saline (PBS) into the left wing patagium. Down feathers were removed from the patagium prior to the injection. Patagium thickness was measured (± 0.01 mm) with a pressure-sensitive micrometer (Mitutoyo, Kawasaki, Japan) immediately before and 24h after injection. Each measurement was replicated three times and averaged. The mean difference of average thickness before and after injection was used as an index of PHA response. Since immunocompetence develops during ontogeny [59], we also examined age-related variation in PHA response. We found evidence for non-linear changes in PHA response with chick age (Table S1). Hence, age-corrected (residual) PHA response values were extracted from the quadratic regression and used in the analysis.

### Post-fledging survival

**Fig. 1:**
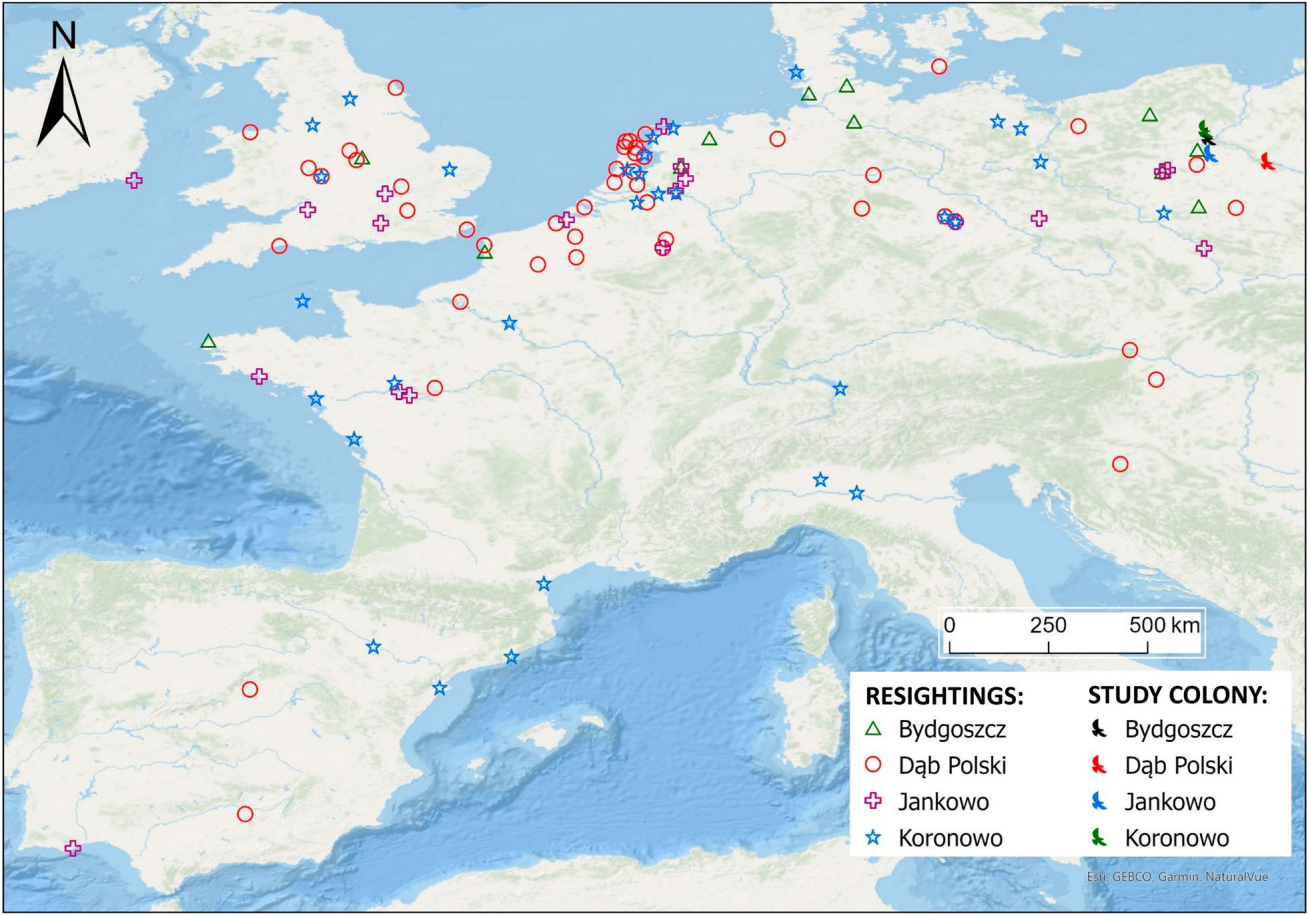
A map showing location of four study colonies of the black-headed gull in Poland and locations of post-fledging (first-year) resightings of marked chicks

To estimate post-fledging survival, we compiled resighting data for our experimental birds, i.e. individuals for which immune response was experimentally induced and quantified. Resighting data were provided by the Ornithological Station of the Museum and Institute of Zoology Polish Academy of Sciences (PAS), which coordinates bird ringing and archives all bird ringing records from Poland together with all their national and international resightings. Here, we focused on resightings of live individuals during their first year of life, as this period primarily reflects post-fledging dispersal movements of young gulls prior to recruitment. In total, we retrieved resightings from 110 (20.8%) birds, originating mainly from the Netherlands, Belgium and Great Britain (Fig. 1). These areas are consistent with the general directions of seasonal movements of the Central European populations of the black-headed gull [60], suggesting no major spatial biases in our resighting data. Despite this, we acknowledge that resighting effort could exhibit some local geographical variation. Resightings of each individual were assigned into one-month intervals starting from the first month after hatching and used to construct encounter histories comprising twelve encounter occasions, where the first occasion corresponded to the marking event.

### Statistical analyses

To estimate post-fledging survival, we applied Cormack-Jolly-Seber (CJS) models based on the framework outlined by Lebreton et al. [61]. CJS models simultaneously estimate encounter probability *p* (the probability of resighting a marked individual) and survival probability, which typically corresponds to apparent (local) survival (φ) that is a product of true survival (S) and site fidelity (increasing with increasing study area [62]). However, in our study, the estimated survival probability represents true survival (S), as the study (resighting) area was virtually unlimited and resightings originated from the entire range of the study population, so the permanent emigration was effectively absent. While the estimated true survival represents percentage of birds that survive from one encounter occasion (month) to another, encounter (resighting) probability represents percentage of surviving individuals that are detected on each encounter occasion (meaning that undetected individuals may be either alive or dead). Models originally implemented in MARK [63] were run using the *RMark* R package [64] developed for the R statistical environment (R Foundation for Statistical Computing, Vienna, Austria).

We first fitted models in which survival (S) was either constant (null model) or varied as a function of time (i.e. between successive resighting periods), year, or colony. This approach yielded one null model and seven models with all possible combinations of three basic predictors. Next, each of these models was extended with a single additional predictor (either hatching date, chick PHA response or chick size-corrected body mass), resulting in 24 additional models. Combination of these additional predictors were not included in the models due to multicollinearity (body condition and immunocompetence changed with hatching date; see Results for details). Also, we did not include chick blood haemoglobin concentration as an additional predictor due to lower sample size (17.5% missing values). Encounter probability (*p*) was modelled as either constant or time-dependent, producing a total of 64 alternative models fitted. All models were ranked using the Akaike Information Criterion corrected for small sample size (AIC_C_) [65]. Model rankings were generated using the *collect.models* function in *RMark*. The best-fitting model was used to estimate post-fledging survival rate.

We also used general linear mixed models (GLMMs) to analyse intra- and inter-seasonal variation in chick body condition and immunocompetence. Size-corrected body mass, age-corrected blood haemoglobin concentration, and age-corrected PHA response were entered as response variables in separate models, with hatching date and year included as predictors. Colony was entered as a random factor in each model. All GLMMs were fitted using the restricted maximum likelihood (REML) approach, as implemented in the *lme4* R package [66]. Marginal and conditional model R^2^ values, reflecting the proportion of variance explained by fixed factors only (marginal) or by both fixed and random factors (conditional), were calculated according to Nakagawa et al. [67] using the *partR2* R package [68]. Pairwise correlations between chick body condition and immunocompetence were tested using Pearson’s product-moment correlation coefficient. All values are reported as means ± SE.

## Results

**Fig. 2:**
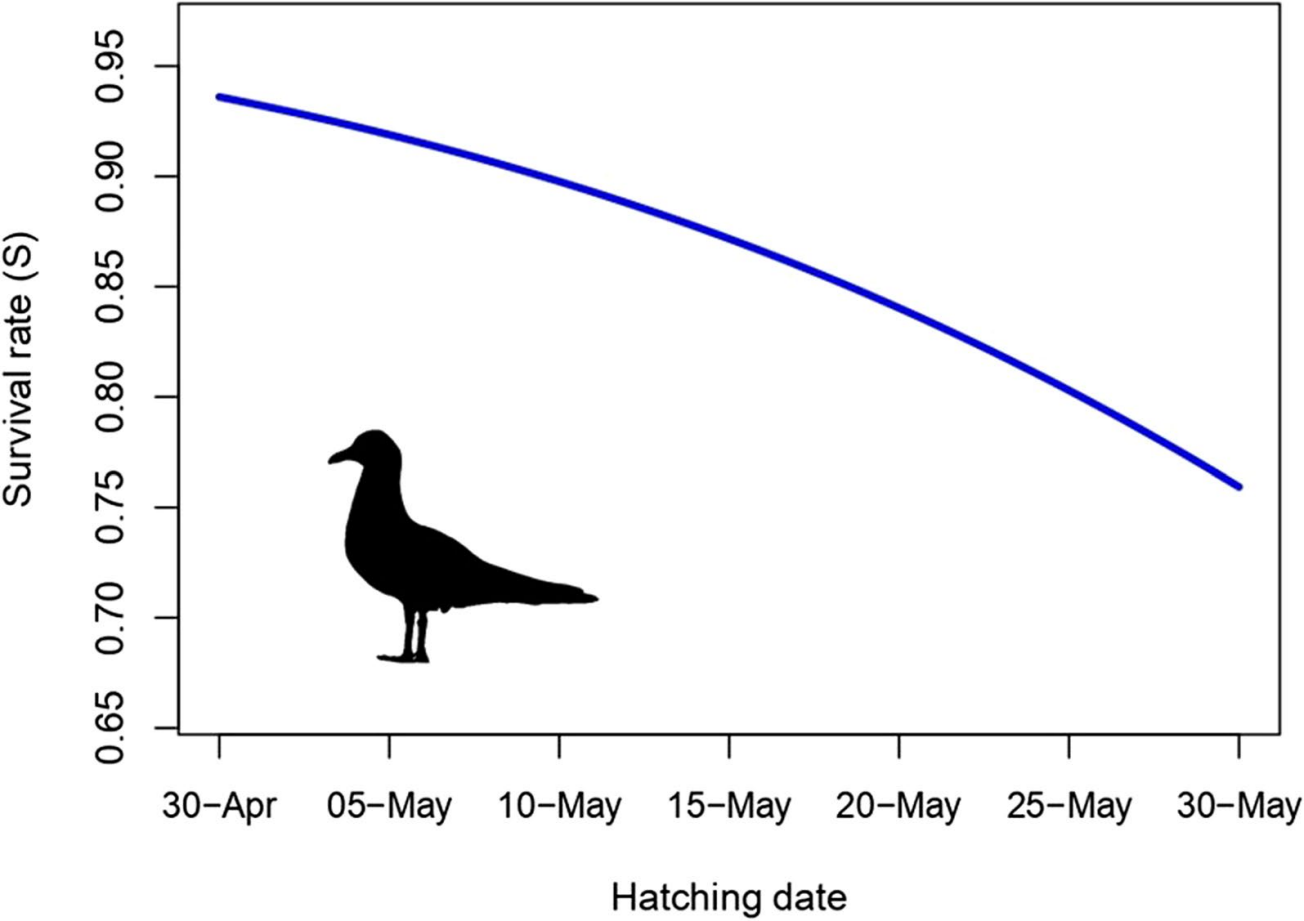
Association of monthly post-fledging survival rate (first year of life) with hatching date in black-headed gulls

The estimated monthly post-fledging survival rate of black-headed gulls was S = 0.89 ± 0.02 under the null capture-recapture model (constant survival across years and colonies), corresponding to an annual survival rate of S = 0.28 during the first year of life. The best-fitting model (no. 1.1. in Table 1) indicated that survival rate varied among years (S = 0.80–0.94; Table S2) and was lower in later-hatched chicks compared to early hatched chicks (Fig. 2). There was no support for effects of chick body condition or immunocompetence on post-fledging survival (all models with ΔAICc > 2; Table S3). There was also no support for differences in post-fledging survival among colonies (Table S3). The estimated monthly resighting probability in the null model was 0.04, but the best-fitting model revealed temporal variation in this parameter between resighting periods (0.02–0.10; Table S2).

**Table 1:** Best-fitting (ΔAIC_C_ < 2) capture-recapture models of post-fledging survival in the first year of life in black-headed gulls. S refers to the monthly true survival probability, while p refers to the monthly resighting probability. The null model was added as a reference. Model subscripts: (.)–constant, t–time (resighting periods), yr–year, h_date–hatching date. N_P_ stands for the number of parameters

Model no	Model	N_p_	AICc	ΔAICC	Weight	Deviance
1	S(yr + h_date), p(t)	15	1163.30	0.00	0.23	1132.53
2	S(yr), p(t)	14	1163.99	0.68	0.17	132.10
3	S(yr + h_date), p(.)	5	1164.35	1.04	0.14	1154.25
4	S(yr), p(.)	4	1165.17	1.87	0.09	153.89
null	S(.), p(.)	2	1176.48	13.17	0.00	169.25

**Fig. 3:**
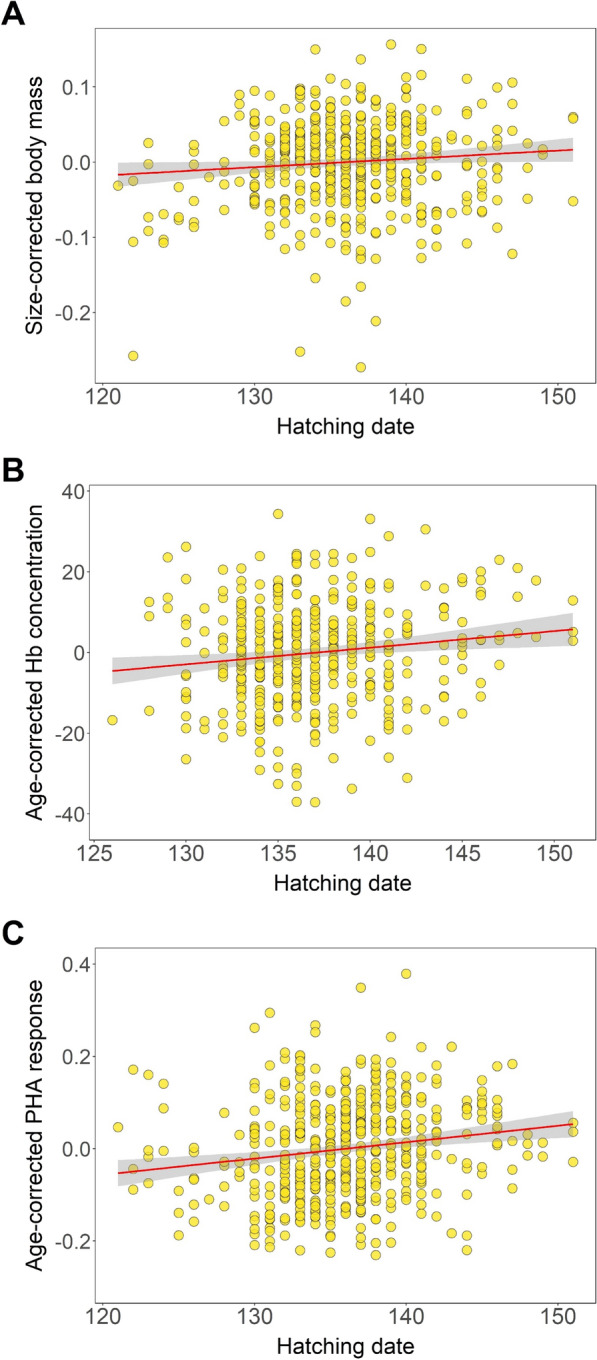
Associations of condition and immune response with hatching date in black-headed gull chicks. Regression lines (red) with 95% confidence intervals (grey) are shown. Size-corrected body mass was quantified as residuals from the log-quadratic regression of chick body mass against wing length, while age-corrected haemoglobin (Hb) concentration and PHA were quantified as residuals from the quadratic regressions of chick total blood Hb concentration and PHA response against age (days). Hatching day was expressed as the day of the year

We found that chick body condition increased with delayed hatching date, as late-hatched chicks had significantly higher size-corrected body mass (β = 0.0010 ± 0.0005, p = 0.041) and total blood haemoglobin concentration (β = 0.3286 ± 0.1527, p = 0.032) compared to early-hatched chicks (Table 2, Fig. 3). The PHA response also increased significantly with delayed hatching date, indicating stronger immune defence in late-hatched chicks (β = 0.0027 ± 0.0010, p = 0.005, Table 2). The full models explained 7.6–38.6% of the variance in chick condition and immunocompetence. No significant pairwise correlations were found between body condition and immunocompetence (all p > 0.500).

**Table 2:** General linear mixed models assessing intra- and inter-seasonal variation in chick condition and immune traits of the black-headed gull. Colony was entered as a random factor in each model. Significant predictors are marked in bold

Trait	Predictor	β	SE	T	P
Size-corrected body mass	Intercept	−0.1513	0.0822	1.84	0.076
	**Hatching date**	**0.0010**	**0.0005**	**2.03**	**0.041**
	Year (2021)	0.0246	0.0477	0.52	0.659
	Year (2022)	0.0034	0.0478	0.07	0.950
		*Marginal R*^*2*^ = *0.029*		*Conditional R*^*2*^ = *0.386*	
Blood haemoglobin concentration	**Intercept**	**−43.729**	**21.310**	**2.05**	**0.041**
	**Hatching date**	**0.3286**	**0.1527**	**2.15**	**0.032**
	Year (2021)	−2.7149	3.6932	0.74	0.549
	Year (2022)	−2.507	3.7401	0.67	0.578
		*Marginal R*^*2*^ = *0.025*		*Conditional R*^*2*^ = *0.076*	
PHA response	**Intercept**	**−0.3448**	**0.1332**	**2.59**	**0.010**
	**Hatching date**	**0.0027**	**0.0010**	**2.84**	**0.005**
	Year (2021)	−0.0246	0.0262	0.94	0.499
	Year (2022)	−0.0451	0.0266	1.69	0.299
		*Marginal R*^*2*^ = *0.056*		*Conditional R*^*2*^ = *0.094*

## Discussion

The results of this study showed that better early-life body condition and immunocompetence were not associated with higher post-fledging survival in black-headed gulls. On the contrary, we found contrasting associations of early-life phenotypic quality and post-fledging survival with respect to hatching date. Specifically, late-hatched chicks showed higher body condition and immunocompetence, but lower post-fledging survival than those hatched earlier in the year. Our results also indicate that the timing of development is a better predictor of post-fledging survival than early-life phenotypic quality.

Our capture-recapture analysis of extensive resighting data from black-headed gulls during the first year of life clearly indicated that earlier hatching date promoted higher post-fledging survival. Associations of hatching or fledging dates with post-fledging survival were previously reported for diverse avian taxa. Earlier hatching and fledging was associated with enhanced first-year survival in large non-passerine birds, such as the European shag *Gulosus aristotelis* [69] and white stork *Ciconia ciconia* [42]. There was also evidence for post-fledging survival declining with hatching date in colonial larid species, including the roseate tern *Sterna dougallii* [70] and herring gull *Larus argentatus* [41]. However, research on model passerine bird species, such as great tits *Parus major,* provided evidence for the complex associations of post-fledging survival and the timing of development. In the Dutch great tit population, chicks fledged early in the breeding season had higher recruitment rate than late fledglings [71], but either early, intermediate or late fledged great tits from a Spanish population showed the highest recruitment in different years [39]. In the great tits and coal tits *Periparus ater* from Switzerland juvenile survival was enhanced by a combination of early fledging and higher chick condition at fledging, with a stronger positive effect of body condition in late fledged birds [72]. In another passerine species, the Swainson’s thrush *Catharus ustulatus*, fledging date was positively associated with juvenile survival [40].

Breeding phenology in wild birds is generally optimised by interactions between weather conditions and food availability [73, 74]. The mismatch hypothesis predicts that reproductive success of birds breeding in seasonal environments is maximized when reproduction is well synchronized with the temporal peak in food supply [75]. However, while fledging success tends to increase when peak food abundance coincides with peak energy demands of the brood, such synchronization may not necessarily be critical for post-fledging performance. In fact, long-term survival may depend less on environmental conditions and parental investment during the rearing period, but more on the conditions experienced immediately after fledging, when young birds become independent [76]. Optimal environmental conditions, especially high food abundance, during this critical period can promote development of efficient foraging and competitive skills, enhance predator avoidance, and facilitate fat accumulation required for energetically demanding processes, such as the first autumn migration and moult. Our research on black-headed gulls indicates that early hatching promotes higher post-fledging survival, despite having negative effects on chick phenotypic quality (body condition and immunocompetence). Early-hatched chicks typically fledge around late May and their early period of independence may be well synchronised with seasonal peak in food abundance, possibly contributing to their higher post-fledgling survival. The diet of black-headed gulls is relatively diverse, including earthworms, insects and small vertebrates (e.g. fish) supplemented with seeds and plant material [77], so temporal fluctuations in prey availability are difficult to predict. Consequently, we acknowledge that the synchronization scenario is only speculative, but it nevertheless provides a possible explanation of how disadvantages resulting from the lower phenotypic quality of early hatched chicks can be mitigated.

Late hatching may not only lead to suboptimal synchronisation between the period of independence and peak food availability, but also impose substantial constraints on later stages of the annual cycle. Specifically, despite their higher phenotypic quality, late-hatched chicks may have insufficient time to adequately prepare for migration [78]. Late-hatched chicks fledge in July or even later and they may not fully develop flight and foraging abilities before departure, possibly leading to greater energy expenditure during migration, more frequent stopovers, delayed arrival at wintering grounds, and consequently, competitive disadvantages in securing optimal wintering sites. Indeed, there is accumulating evidence that early-life activity is related to skill acquisition [79, 80], which may be of key importance for migrating birds that face strong selection shortly after fledging during their first autumn migration [81, 82]. Such cascading effects are consistent with the so-called ‘domino effect’, in which suboptimal timing of one event triggers a sequence of delays across subsequent life-cycle stages [83]. We therefore suggest that the cumulative negative effects of delayed hatching and fledging in black-headed gulls may outweigh any benefits of favourable rearing conditions, ultimately compromising post-fledging survival. Consistent with this hypothetical scenario, studies on other bird species indicate that temporal mismatches across different annual stages may represent potential bottlenecks for survival [84, 85], and thus temporal synchronization appears to be among the important determinants of avian fitness.

There is also accumulating evidence from studies of wild birds that events occurring at one stage of life carry over to affect individual performance at subsequent stages [86]. For example, a shortened breeding season has been shown to result in poorer condition prior to autumn migration in savannah sparrows *Passerculus sandwichensis* [32]. A similar pattern has been described in the kittiwake, where a longer parental rearing period enhanced offspring condition at independence, readiness for migration, and first-winter survival [36]. Evidence from several migratory species indicates that early-hatched juveniles have more time to develop morphologically and accumulate fat reserves before the onset of autumn migration, which facilitates earlier migration [32, 64, 86, 87]. In blue tits *Cyanistes caeruleus*, both hatching date and condition predicted subcutaneous fat levels during autumn migration [88], indicating that the timing of reproduction and rearing conditions (e.g. parental investment) both determine post-fledging performance. Other studies, however, point to the leading role of chick phenotypic quality rather than the timing of development, as a predictor of future performance and fitness. For instance, recruitment rate of pied flycatchers *Ficedula hypoleuca* was primarily determined by the strength of PHA response during the natal stage, but not hatching date [35]. Such findings clearly contrast with our current results, in which breeding phenology emerges as a more important determinant of post-fledging survival than chick phenotypic quality.

Although our findings contribute to the understanding of long-term consequences of early-life conditions in the black-headed gull, we still need to acknowledge certain limitations of our research framework. Notably, our study is of correlative nature, so any mechanistic explanations for associations between hatching date and post-fledging survival or chick phenotypic quality are only speculative. While we treat seasonal variation in food availability as the most plausible explanation, we have no empirical evidence to support this explanation, and other scenarios cannot be excluded. For instance, we have no information of parental quality, which may also show temporal variation over the course of the breeding season, as described in other colonial waterbirds [89–92]. Variation in the breeding phenology by parents of different quality could possibly contribute to associations between offspring phenotypic quality, the timing of development, and post-fledging survival. We recommend that experimental manipulation of either parental quality or food availability is necessary to provide causal resolution of these associations in the future.

## Conclusions

Our study failed to find any association between chick phenotypic quality (body condition and immunocompetence) and post-fledging survival in the black-headed gull. Instead, our analyses pointed to breeding phenology as a key determinant of post-fledging survival rates, as early hatched chicks had enhanced survival. Although our data did not allow us to unambiguously identify mechanisms underlying this association, we suggest that it could possibly be driven by the temporal synchronization between the seasonal peak in food availability and critical stages of the post-fledging period. We recommend that future studies endeavour to determine the relative importance of pre- and post-fledging body condition/immunocompetence on future performance in wild birds. We are, though, aware that obtaining extensive data on phenotypic quality of independent juveniles during the post-fledging period may be technically challenging. While further experimental research is necessary to better understand the mechanisms underlying carry-over effects across avian life cycles, our study suggests that long-term fitness consequences of early life may be complex and more dependent on the timing of development rather than phenotypic quality during the natal stage.

## Supplementary Information

Additional file1 (DOCX 27 KB)

## Data Availability

The datasets used and/or analysed during the current study are available from the corresponding author on reasonable request.
