# Supplementary material for: Earlier hatching date rather than chick phenotypic quality enhances first-year survival in the black-headed gull
Source: Front Zool. 2026 Mar 17;23:16. doi: 10.1186/s12983-026-00605-6 (PMC13107678; doi:10.1186/s12983-026-00605-6)
Supplement: Supplementary file 1 — Additional file1 (DOCX 27 KB) [file 12983_2026_605_MOESM1_ESM.docx]

**Electronic Supplementary Material**

**Earlier hatching date rather than chick phenotypic quality enhances first-year survival in the black-headed gull**

Piotr Indykiewicz^1^, Julia Barczyk^2^, Jarosław Kowalski^3^, and Piotr Minias^4^*

^1^ UTP University of Science and Technology, Faculty of Animal Breeding and Biology, Department of Biology and Animal Environment, Mazowiecka 28, 85-084 Bydgoszcz, Poland; ORCID 0000-0003-1254-2424.

^2^ University of Wrocław, Faculty of Biological Sciences, Department of Avian Ecology, Sienkiewicza 21, 50-335 Wrocław, Poland; ORCID: 0000-0002-9387-2179.

^3^ Gorzewo 7, 09-200, Sierpc, Poland; ORCID: 0000-0003-2708-5887.

^4^ University of Lodz, Faculty of Biology and Environmental Protection, Department of Biodiversity Studies and Bioeducation, , Banacha 1/3, 90-237 Lódź, Poland; ORCID: 0000-0002-7742-6750.

*Correspondence:

Piotr Minias

pminias@op.pl

**Table S1.** Regression models assessing age-related variation in nestling condition and immune traits of the black-headed gull. Significant predictors are marked in bold.

| Trait | Predictor | β | SE | t | P |
| --- | --- | --- | --- | --- | --- |
| Size-corrected body mass | Intercept | -0.010 | 0.012 | 0.86 | 0.391 |
|  | Age | 0.0006 | 0.0014 | 0.45 | 0.652 |
|  | Age squared | -0.000004 | 0.000037 | 0.10 | 0.919 |
|  |  |  | | *Marginal R^2^ = 0.005* | |
| Blood haemoglobin concentration | **Intercept** | **-80.459** | **2.894** | **27.80** | **<0.001** |
|  | **Age** | **2.918** | **0.336** | **8.69** | **<0.001** |
|  | **Age squared** | **-0.055** | **0.009** | **6.31** | **<0.001** |
|  |  |  | | *Marginal R^2^ = 0.280* | |
| PHA response | **Intercept** | **0.097** | **0.023** | **4.30** | **<0.001** |
|  | **Age** | **0.013** | **0.003** | **5.18** | **<0.001** |
|  | **Age squared** | **-0.00033** | **0.00007** | **4.88** | **<0.001** |
|  |  |  | | *Marginal R^2^ = 0.050* | |

|  |
| --- |

**Table S2.** Best-fitting model (ΔAIC_c_ = 0) estimates of monthly true survival (S) and resighting probability (p) in the black headed gulls during the post-fledging period (first year of life).

| Predictor | Estimate | SE | Lower confidence limit | Upper confidence limit |
| --- | --- | --- | --- | --- |
| S (2019) | 0.94 | 0.04 | 0.81 | 0.98 |
| S (2021) | 0.85 | 0.05 | 0.73 | 0.92 |
| S (2022) | 0.80 | 0.05 | 0.69 | 0.87 |
| p (t2) | 0.06 | 0.01 | 0.04 | 0.09 |
| p (t3) | 0.05 | 0.01 | 0.03 | 0.08 |
| p (t4) | 0.02 | 0.01 | 0.01 | 0.04 |
| p (t5) | 0.02 | 0.01 | 0.01 | 0.05 |
| p (t6) | 0.04 | 0.01 | 0.02 | 0.08 |
| p (t7) | 0.05 | 0.02 | 0.02 | 0.10 |
| p (t8) | 0.03 | 0.02 | 0.01 | 0.09 |
| p (t9) | 0.05 | 0.02 | 0.02 | 0.12 |
| p (t10) | 0.06 | 0.03 | 0.02 | 0.15 |
| p (t11) | 0.03 | 0.02 | 0.01 | 0.10 |
| p (t12) | 0.10 | 0.05 | 0.03 | 0.25 |

**Table S3.** Capture-recapture models of post-fledging survival (first year of life) in the black-headed gulls. S refers to monthly true survival probability, while p refers to monthly resighting probability. Model subscripts: (.) – constant, t – time (resighting periods), yr – year, col – colony, h_date – hatching date, bm – nestling residual size-corrected body mass, PHA – nestling residual PHA response. Np stands for the number of parameters.

| Mode no. | Model | Np | AICc | DeltaAICc | weight | Deviance |
| --- | --- | --- | --- | --- | --- | --- |
| 1 | S(yr + h_date) p(t) | 15 | 1163.30 | 0.00 | 0.22 | 1132.53 |
| 2 | S(yr) p(t) | 14 | 1163.99 | 0.68 | 0.16 | 156.25 |
| 3 | S(yr + h_date) p(.) | 5 | 1164.35 | 1.04 | 0.13 | 1154.25 |
| 4 | S(yr) p(.) | 4 | 1165.17 | 1.87 | 0.09 | 178.04 |
| 5 | S(yr + bm) p(t) | 15 | 1165.62 | 2.31 | 0.07 | 1134.85 |
| 6 | S(yr + PHA) p(t) | 15 | 1165.62 | 2.32 | 0.07 | 1134.85 |
| 7 | S(yr + PHA) p(.) | 5 | 1166.60 | 3.30 | 0.04 | 1156.51 |
| 8 | S(yr + bm) p(.) | 5 | 1166.65 | 3.35 | 0.04 | 1156.56 |
| 9 | S(t + yr + h_date) p(.) | 15 | 1167.65 | 4.34 | 0.02 | 1136.88 |
| 10 | S(col + h_date) p(t) | 17 | 1167.76 | 4.46 | 0.02 | 1132.78 |
| 11 | S(col) p(t) | 16 | 1167.83 | 4.52 | 0.02 | 155.89 |
| 12 | S(col + h_date) p(.) | 7 | 1168.78 | 5.48 | 0.01 | 1154.60 |
| 13 | S(col) p(.) | 6 | 1168.85 | 5.55 | 0.01 | 177.66 |
| 14 | S(col + bm) p(t) | 17 | 1168.95 | 5.65 | 0.01 | 1133.97 |
| 15 | S(t + yr) p(.) | 14 | 1169.15 | 5.85 | 0.01 | 161.42 |
| 16 | S(col + PHA) p(t) | 17 | 1169.43 | 6.13 | 0.01 | 1134.45 |
| 17 | S(col + bm) p(.) | 7 | 1169.74 | 6.44 | 0.01 | 1155.56 |
| 18 | S(col + PHA) p(.) | 7 | 1170.24 | 6.94 | 0.01 | 1156.06 |
| 19 | S(t + yr + PHA) p(.) | 15 | 1170.45 | 7.15 | 0.01 | 1139.68 |
| 20 | S(t + yr + bm) p(.) | 15 | 1170.75 | 7.44 | 0.01 | 1139.98 |
| 21 | S(col + yr + h_date) p(t) | 19 | 1171.63 | 8.32 | 0.00 | 1132.40 |
| 22 | S(yr + col) p(t) | 18 | 1172.03 | 8.73 | 0.00 | 155.87 |
| 23 | S(col + yr + h_date) p(.) | 9 | 1172.40 | 9.10 | 0.00 | 1154.11 |
| 24 | S(t + col + h_date) p(.) | 17 | 1172.45 | 9.14 | 0.00 | 1137.46 |
| 25 | S(yr + col) p(.) | 8 | 1172.92 | 9.61 | 0.00 | 177.63 |
| 26 | S(t + col) p(.) | 16 | 1173.04 | 9.74 | 0.00 | 161.11 |
| 27 | S(col + yr + bm) p(t) | 19 | 1173.18 | 9.88 | 0.00 | 1133.96 |
| 28 | S(col + yr + PHA) p(t) | 19 | 1173.67 | 10.37 | 0.00 | 1134.45 |
| 29 | S(col + yr + bm) p(.) | 9 | 1173.84 | 10.53 | 0.00 | 1155.55 |
| 30 | S(t + col + bm) p(.) | 17 | 1173.90 | 10.59 | 0.00 | 1138.91 |
| 31 | S(.) p(t) | 12 | 1174.05 | 10.75 | 0.00 | 170.50 |
| 32 | S(t + col + PHA) p(.) | 17 | 1174.18 | 10.88 | 0.00 | 1139.20 |
| 33 | S(col + yr + PHA) p(.) | 9 | 1174.35 | 11.04 | 0.00 | 1156.06 |
| 34 | S(t + yr + h_date) p(t) | 25 | 1174.57 | 11.27 | 0.00 | 1122.46 |
| 35 | S(PHA) p(t) | 13 | 1174.94 | 11.63 | 0.00 | 1148.36 |
| 36 | S(h_date) p(t) | 13 | 1175.73 | 12.43 | 0.00 | 1149.15 |
| 37 | S(t + col + yr + h_date) p(.) | 19 | 1175.92 | 12.61 | 0.00 | 1136.69 |
| 38 | S(bm) p(t) | 13 | 1176.14 | 12.83 | 0.00 | 1149.56 |
| 39 | S(t + yr) p(t) | 24 | 1176.45 | 13.15 | 0.00 | 147.45 |
| 40 (null) | S(.) p(.) | 2 | 1176.48 | 13.17 | 0.00 | 193.40 |
| 41 | S(t + col + yr) p(.) | 18 | 1177.07 | 13.77 | 0.00 | 160.91 |
| 42 | S(PHA) p(.) | 3 | 1177.15 | 13.85 | 0.00 | 1171.12 |
| 43 | S(t + yr + PHA) p(t) | 25 | 1177.84 | 14.54 | 0.00 | 1125.72 |
| 44 | S(t + yr + bm) p(t) | 25 | 1177.99 | 14.68 | 0.00 | 1125.87 |
| 45 | S(h_date) p(.) | 3 | 1178.00 | 14.70 | 0.00 | 1171.96 |
| 46 | S(t + col + yr + bm) p(.) | 19 | 1178.09 | 14.79 | 0.00 | 1138.87 |
| 47 | S(t + col + yr + PHA) p(.) | 19 | 1178.36 | 15.05 | 0.00 | 1139.13 |
| 48 | S(bm) p(.) | 3 | 1178.50 | 15.19 | 0.00 | 1172.46 |
| 49 | S(t + col + h_date) p(t) | 27 | 1178.88 | 15.58 | 0.00 | 1122.42 |
| 50 | S(t) p(.) | 12 | 1180.17 | 16.87 | 0.00 | 176.61 |
| 51 | S(t + col) p(t) | 26 | 1180.27 | 16.97 | 0.00 | 146.92 |
| 52 | S(t + PHA) p(.) | 13 | 1180.47 | 17.16 | 0.00 | 1153.89 |
| 53 | S(t + col + bm) p(t) | 27 | 1181.28 | 17.97 | 0.00 | 1124.81 |
| 54 | S(t + col + PHA) p(t) | 27 | 1181.64 | 18.33 | 0.00 | 1125.17 |
| 55 | S(t + h_date) p(.) | 13 | 1181.64 | 18.33 | 0.00 | 1155.06 |
| 56 | S(t + bm) p(.) | 13 | 1182.26 | 18.95 | 0.00 | 1155.68 |
| 57 | S(t + col + yr + h_date) p(t) | 29 | 1183.05 | 19.75 | 0.00 | 1122.20 |
| 58 | S(t + col + yr) p(t) | 28 | 1184.63 | 21.33 | 0.00 | 146.92 |
| 59 | S(t + col + yr + bm) p(t) | 29 | 1185.63 | 22.33 | 0.00 | 1124.78 |
| 60 | S(t + col + yr + PHA) p(t) | 29 | 1186.02 | 22.71 | 0.00 | 1125.17 |
| 61 | S(t + PHA) p(t) | 23 | 1191.45 | 28.15 | 0.00 | 1143.67 |
| 62 | S(t) p(t) | 22 | 1192.41 | 29.11 | 0.00 | 167.71 |
| 63 | S(t + h_date) p(t) | 23 | 1193.92 | 30.61 | 0.00 | 1146.13 |
| 64 | S(t + bm) p(t) | 23 | 1194.56 | 31.25 | 0.00 | 1146.77 |
